# Role of hydrogen peroxide in intra-operative wound preparation based on an in vitro fibrin clot degradation model

**DOI:** 10.1016/j.jpra.2021.04.008

**Published:** 2021-05-14

**Authors:** Zita M. Jessop, Elena García-Gareta, Yadan Zhang, Thomas H. Jovic, Nafiseh Badiei, Vaibhav Sharma, Iain S. Whitaker, Norbert Kang

**Affiliations:** a Reconstructive Surgery & Regenerative Medicine Research Group (ReconRegen), Institute of Life Science, Swansea University, United Kingdom; b The Welsh Centre for Burns and Plastic Surgery, Morriston Hospital, Swansea, United Kingdom; c Regenerative Biomaterials Group, RAFT Institute, Mount Vernon Hospital, Northwood, United Kingdom; d Centre for NanoHealth, Swansea University, United Kingdom; e Department of Plastic and Reconstructive Surgery, Royal Free Hospital, London, United Kingdom

**Keywords:** Wound irrigation, hydrogen peroxide, fibrinolysis, fibrin clot, coagulation assay

## Abstract

Three per cent hydrogen peroxide (H_2_O_2_) is widely used to irrigate acute and chronic wounds in the surgical setting and clinical experience tells us that it is more effective at removing dried-on blood than normal saline alone. We hypothesise that this is due to the effect of H_2_O_2_ on fibrin clot architecture via fibrinolysis. We investigate the mechanisms and discuss the clinical implications using an in vitro model. Coagulation assays with normal saline (NaCl), 1% and 3% concentrations of H_2_O_2_ were performed to determine the effect on fibrin clot formation. These effects were confirmed by spectrophotometry. The effects of 1%, 3% and 10% H_2_O_2_ on the macroscopic and microscopic features of fibrin clots were assessed at set time intervals and compared to a NaCl control. Quantitative analysis of fibrin networks was undertaken to determine the fibre length, diameter, branch point density and pore size. Fibrin clots immersed in 1%, 3% and 10% H_2_O_2_ demonstrated volume losses of 0.09-0.25mm^3^/min, whereas those immersed in the normal saline gained in volume by 0.02±0.13 mm^3^/min. Quantitative analysis showed that H_2_O_2_ affects the structure of the fibrin clot in a concentration-dependent manner, with the increase in fibre length, diameter and consequently pore sizes. Our results support our hypothesis that the efficacy of H_2_O_2_ in cleaning blood from wounds is enhanced by its effects on fibrin clot architecture in a concentration- and time-dependent manner. The observed changes in fibre size and branch point density suggest that H_2_O_2_ is acting on the quaternary structure of the fibrin clot, most likely via its effect on cross-linking of the fibrin monomers and may therefore be of benefit for the removal of other fibrin-dependent structures such as wound slough.

## Introduction

**Figure 1:**
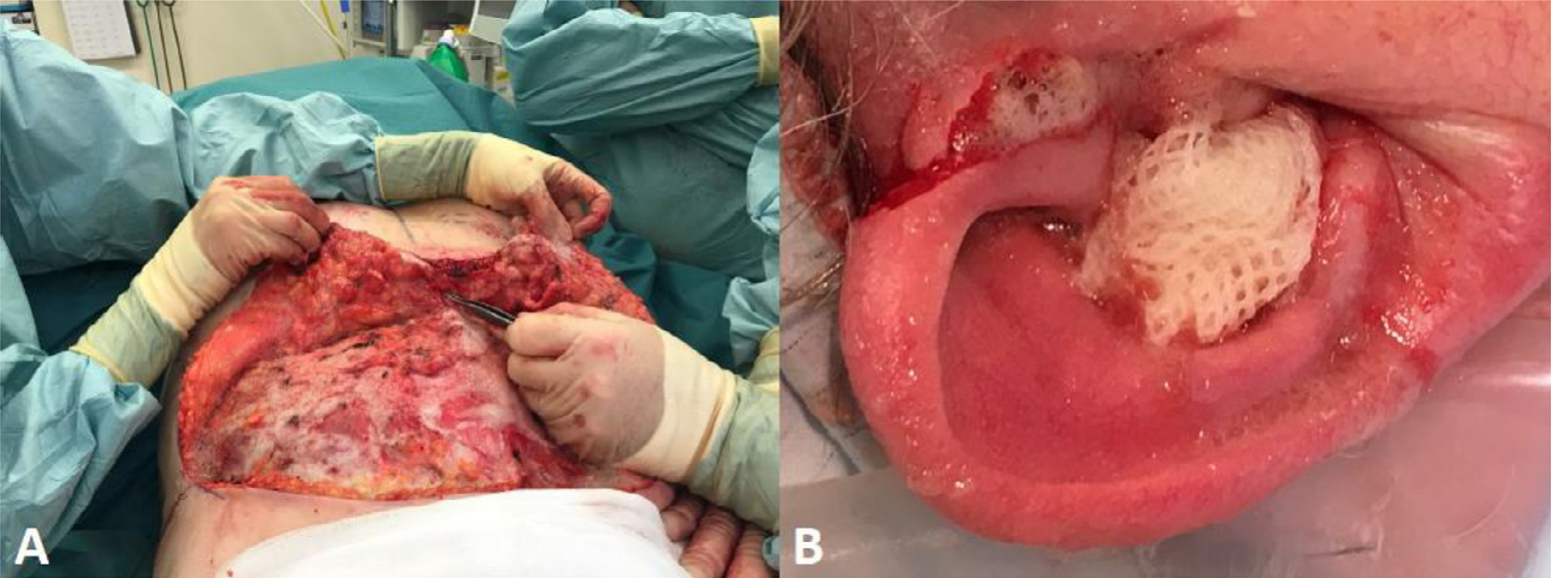
Intraoperative photographs to demonstrate surgical utility of hydrogen peroxide (3% final concentration in a 50:50 mixture with normal saline) in a range of plastic surgical procedures such as abdominoplasty (A) and acute wound debridement of an ear laceration (B).

Hydrogen peroxide (H_2_O_2_), at 3% final concentration in a 50:50 mixture with normal saline (NaCl), is commonly used for irrigating traumatic wounds in a range of plastic and reconstructive surgical procedures (e.g., hand surgery, breast reduction, abdominoplasty, lymph node dissections, acute and chronic wound debridement) (Figure 1). We have also noted that it is very effective for cleaning off clotted and dried-on blood from skin surfaces at the end of a procedure, compared with NaCl irrigation alone. This is often attributed to the effervescence, which can aid in mechanical wound debridement. It can also be helpful with haemostasis by helping to show bleeding points more clearly. For example, there are reports in the literature of the use of H_2_O_2_ to clear blood clots to visualise the base of ulcers during endoscopy.^1^, ^2^, ^3^ However, as with much of surgical practice, the mechanism of action underpinning these clinical observations has never been investigated.

What is known is that a 3% solution of H_2_O_2_ has broad-spectrum bactericidal activity^4^ thought to occur through multiple pathways, including DNA damage,^5^ oxidation of proteins and membrane lipids^6^ and reducing biofilm formation.^7^ However, the use of H_2_O_2_ is not without risk and the main concerns pertain to its cytotoxicity towards host tissue^8^, ^9^, ^10^ and potential for air embolism leading to neurological^11^ and cardiac sequalae.^12^ Because of the potential for oxygen gas formation, hydrogen peroxide should therefore not be used in cases of dural compromise, under pressurised injection, or when irrigating smaller closed spaces or cavities.^13^ The absence of any clear understanding of the way in which H_2_O_2_ achieves its cleaning effect, despite its widespread use in surgery for this purpose, made us think that a more detailed investigation was warranted, especially as there is the potential for H_2_O_2_ to do harm.

Under physiological conditions, the coagulation of blood is activated by thrombin, converting soluble plasma fibrinogen into an insoluble fibrin clot which is degraded enzymatically (fibrinolysis). Hydroxyl radicals, produced by poorly chelated iron ions in the circulation, cause unfolding of the fibrinogen polypeptide chains to expose buried hydrophobic epitopes. This converts soluble human fibrinogen into fibrin clots which are resistant to enzymatic degradation.^14^ Polymerised fibrinogen fibres are different from those produced by the enzymatic action of thrombin. Spontaneous aggregation of insoluble hydrophobic protofibrils results in the formation of dense matted deposits, which (when fused with red blood cells) contributes to the resistance of clots to fibrinolytic degradation.^15^ According to the literature, clot formation is prevented by hydrogen peroxide and by certain other oxidising agents which are able to scavenge hydroxyl radicals:^14^

OH + H_2_O_2_ → O_2_ + H_2_O + H^+^

The inhibitory effect of hydrogen peroxide on the formation of dense fibrin clots may explain some of the therapeutic effects of H_2_O_2_ reported in several papers.^16^, ^17^, ^18^, ^19^ However, there has been little or no research on the effect of H_2_O_2_ on established blood clots. Instead, there has been speculation in the previous literature that H_2_O_2_ oxidises the haemoglobin in established clots, thus rendering the pigmented blood clot translucent, which is then thought to help with its dissolution and clearance.^20^, ^21^ However, we were unable to find any prior studies looking specifically at the effect of H_2_O_2_ on fibrin clot architecture. In this article, we investigate the effects of different concentrations of H_2_O_2_ on the macroscopic and microscopic features of fibrin clots in comparison to an NaCl control and perform quantitative analysis of the fibrin networks to determine fibre length, diameter, branch point density and pore size, which are recognised factors in the susceptibility to fibrinolysis.^22^ These findings may help to explain why H_2_O_2_ is perceived clinically to be so effective in removing clots and dried-on blood.

## Methods

### Fibrin clot formation

A coagulation kinetic assay^23^ was performed using 0.5ml of 2% fibrinogen mixed with either 0.1ml of H_2_O_2_ to make up 1% and 3% solutions or equal volumes of a control substance MES/NaCl buffer [2-(N-morpholino)ethane-sulfonic acid – 25g MES and 44g NaCl dissolved in 5 litres of water, with pH adjusted to 7.4 using 5M NaOH and sterile filtered using a Corning integral filtration unit] with 0.01 ml of 1M calcium. The mixture was incubated for 15 min at 37°C. Fibrinogen cleavage and subsequent fibrin clot formation were initiated by the addition of 0.025 ml (0.25 units) of thrombin. The fibrin-clot endpoint was detected by absorbance at 425nm wavelength of light. The MES/NaCl reaction served as a volume control to eliminate differences related to dilution of reagents by the addition of H_2_O_2_.

### Fibrinolysis assay

**Figure 2:**
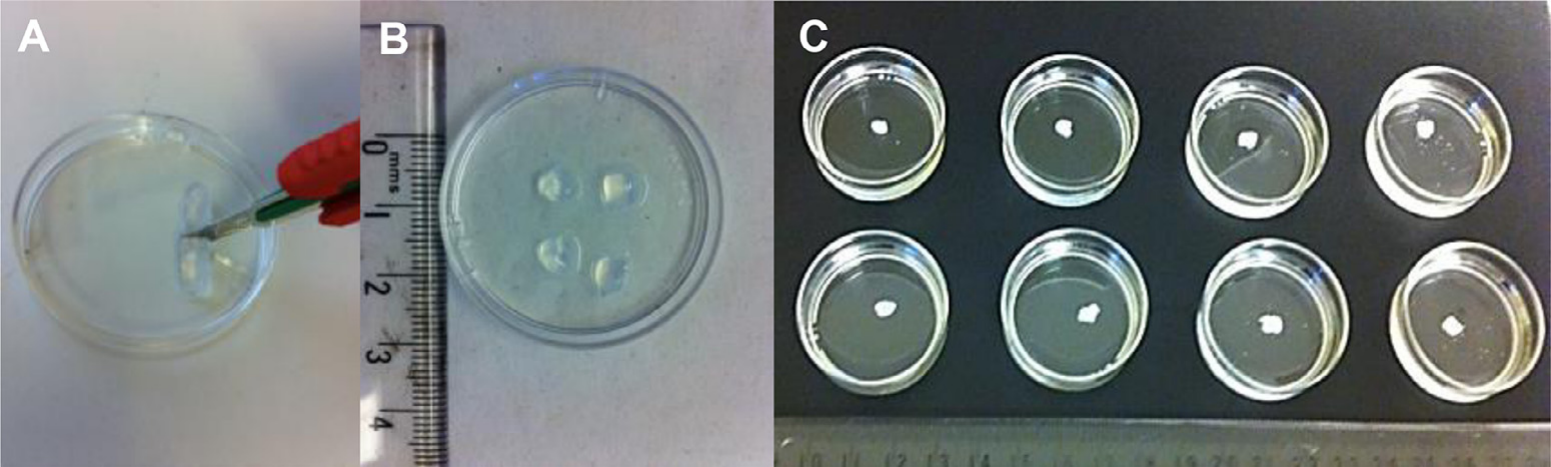
Photographs of fibrinolysis assay preparation: fibrin clots cut to size (A and B), fibrin clots immersed in (left to right) NaCl, 1%, 3% and 10% H_2_O_2_ (C).

Fibrin clots formed by the method described previously in the control group (with no prior H_2_O_2_) were cut to approximately 5 mm^2^ size blocks (Figure 2). These were immersed and agitated in 1%, 3% and 10% H_2_O_2_ solutions and compared with a NaCl control cleaning solutions, with four repeats per treatment. Photographs were taken at 30-min intervals and fibrin clot volume measurements (mm^3^) were taken at 15-min intervals between 0 and 90 min. After 90 min, the fibrin clots were processed for scanning electron microscopy (SEM) to investigate the structural characteristics.

### Scanning electron microscopy of fibrin clots

Fibrin clot samples were washed three times with 50  mM sodium cacodylate-HCl buffer solution (pH 7.2–7.4, SPI Supplies) at 10- to 20-min intervals to remove excess salt. The samples were fixed overnight in 2% glutaraldehyde (Sigma Aldrich, UK) and dehydrated with a series of graded concentrations (30% to 100%) of ethanol. The dehydrated sample was then rinsed with 50% hexamethyldisilazane (HMDS) solution in 100% ethanol for 10 min in a fume hood and then three times in 100% HMDS and left overnight to dry. The sample was coated with a thin layer of gold (~15 nm) using sputter coating and was imaged by SEM (Hitachi 4800).

### Quantitative characterisations of fibrin networks

Fibre length and diameter were measured from digitised scanning electron micrographs using the ImageJ 1.50i analysis software (Wayne Rasband, National Institutes of Health, USA). The complete length of a fibre was defined as the distance measured along the fibre between its terminal branchpoints. Fibre diameter was measured at the centre of each branch. Fibre branch points (points at which three or more fibres joined together) were marked and counted on the SEM images. Branchpoint densities were calculated by dividing the total number of branchpoints measured in the fibrin network area. Pore diameters were measured at their widest point for all samples.

### Statistical Analysis

All the data shown are representative of 4 replicates unless stated otherwise. The distribution of the data was analysed using the Anderson-Darling test to confirm the normality of the data and differences in variances tested using Bartlett's test (Minitab 18 software). Data that were not normal or of equal distribution were subject to log transformation. One-way analysis of variance (ANOVA) on ranks (SigmaStat 3.5 software) with a Turkey test post-hoc analysis was performed to determine the significance of hydrogen peroxide effect on clot volume over time. The results were considered statistically significant at P < 0.05.

## Results

### Fibrin clot formation

**Figure 3:**
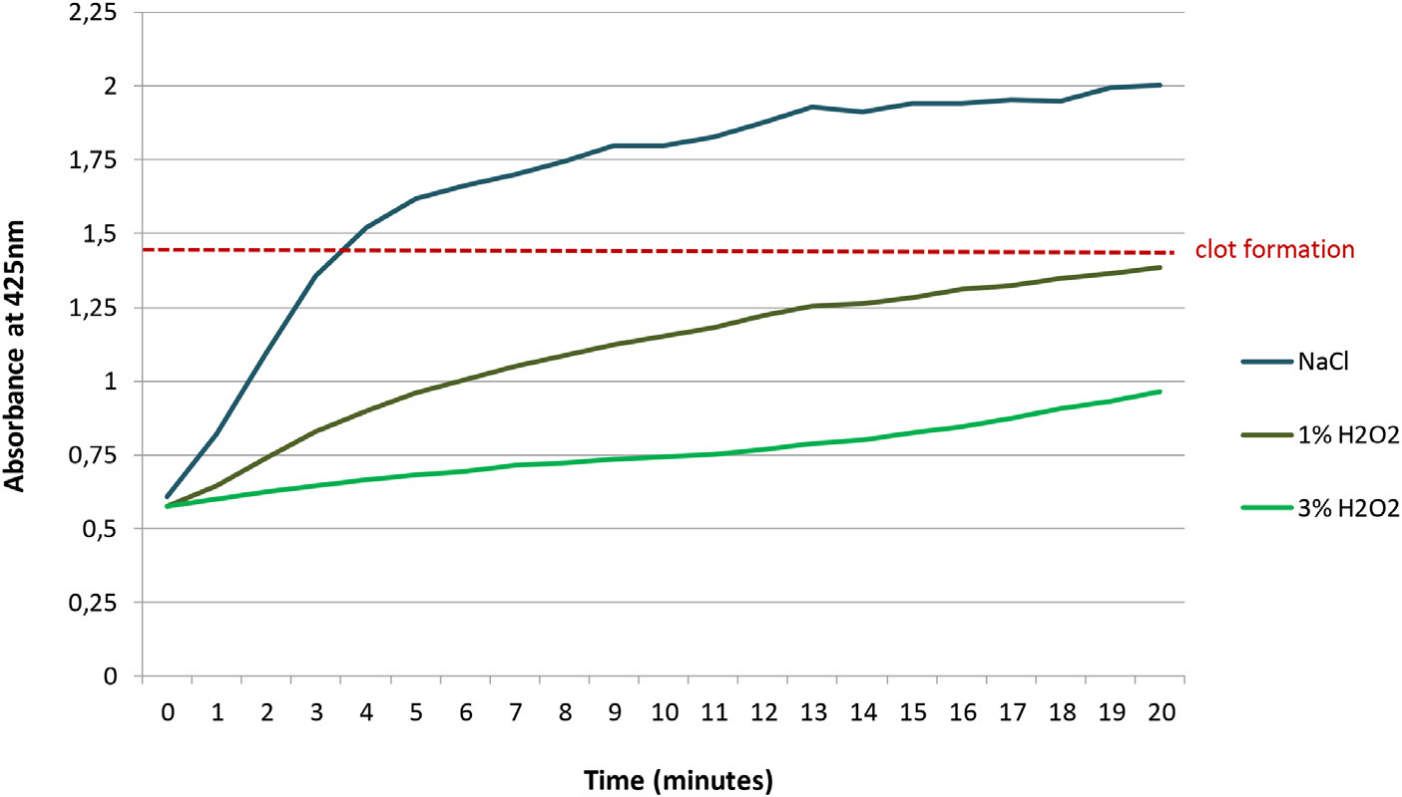
Fibrin clot formation detected by a coagulation kinetic assay carried out in normal saline (NaCl), 1% and 3% H_2_O_2_.

The fibrin-clot endpoint, which was detected by a coagulation kinetic assay using a vis/UV spectrophotometer to measure absorbance at 425nm, is marked by the red dotted line in Figure 3. H_2_O_2_ reduced the rate of initial increase in light absorption, an indicator of fibrin clot formation, in a dose dependent manner (NaCl: 0.20/min; 1% H_2_O_2_: 0.08/min; 3% H_2_O_2_: 0.02/min).

### Fibrinolysis experiments

**Figure 4:**
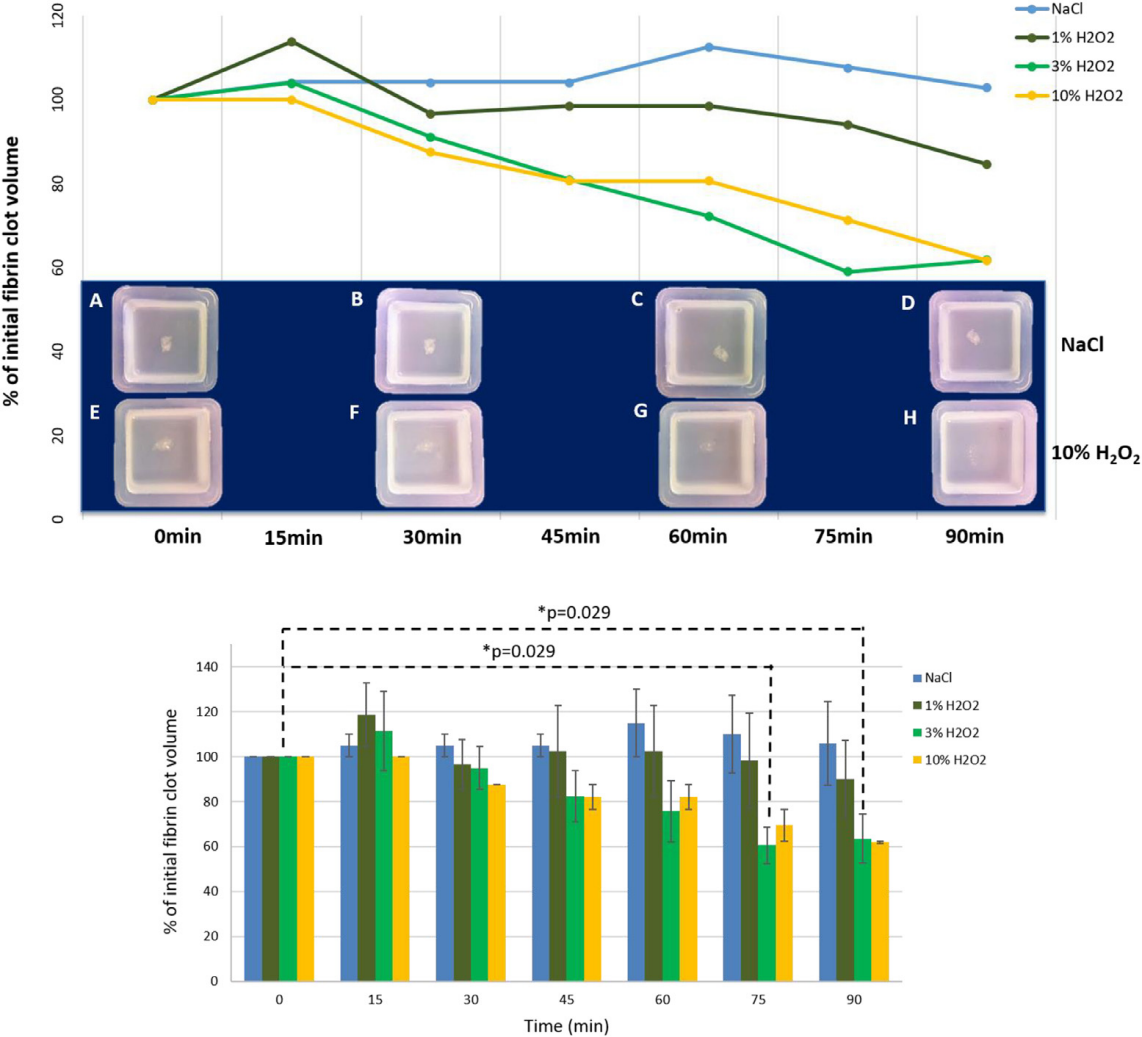
Effect of hydrogen peroxide versus normal saline on fibrin clot volume: graph shows percentage of initial fibrin clot volume over time (results show mean values). Below this graph, macroscopic appearance of fibrin clots agitated in NaCl control (A-D) and 10% H_2_O_2_ (E-H) at 0 (A, E), 30 (B, F), 60 (C, G) and 90 (D, H) minutes can be seen. At the bottom, data are plotted in a bar graph (showing mean ± standard error mean) showing statistical significances.

Fibrin clots immersed in 1%, 3% and 10% H_2_O_2_ demonstrated a 0.09mm^3^/min (±0.14SD), 0.25mm^3^/min (±0.13SD) and 0.14mm^3^/min (±0.04SD) rate of volume loss respectively, whereas those immersed in the NaCl increased in volume by 0.02mm^3^/min (±0.13SD). Photographs also demonstrate increased macroscopic translucency of fibrin clots immersed in 10% H_2_O_2_ (Figure 4E, F, G, H**)** versus NaCl over time (Figure 4A, B, C, D). Statistical analysis showed that after 75min and 90min in 3% H_2_O_2_ there was a significant reduction in clot volume compared to the initial one. No other statistical significance was found although the data suggest a trend: the higher the concentration of H_2_O_2_, the higher was the reduction in clot volume.

### Effect of hydrogen peroxide on fibrin clot architecture

**Figure 5:**
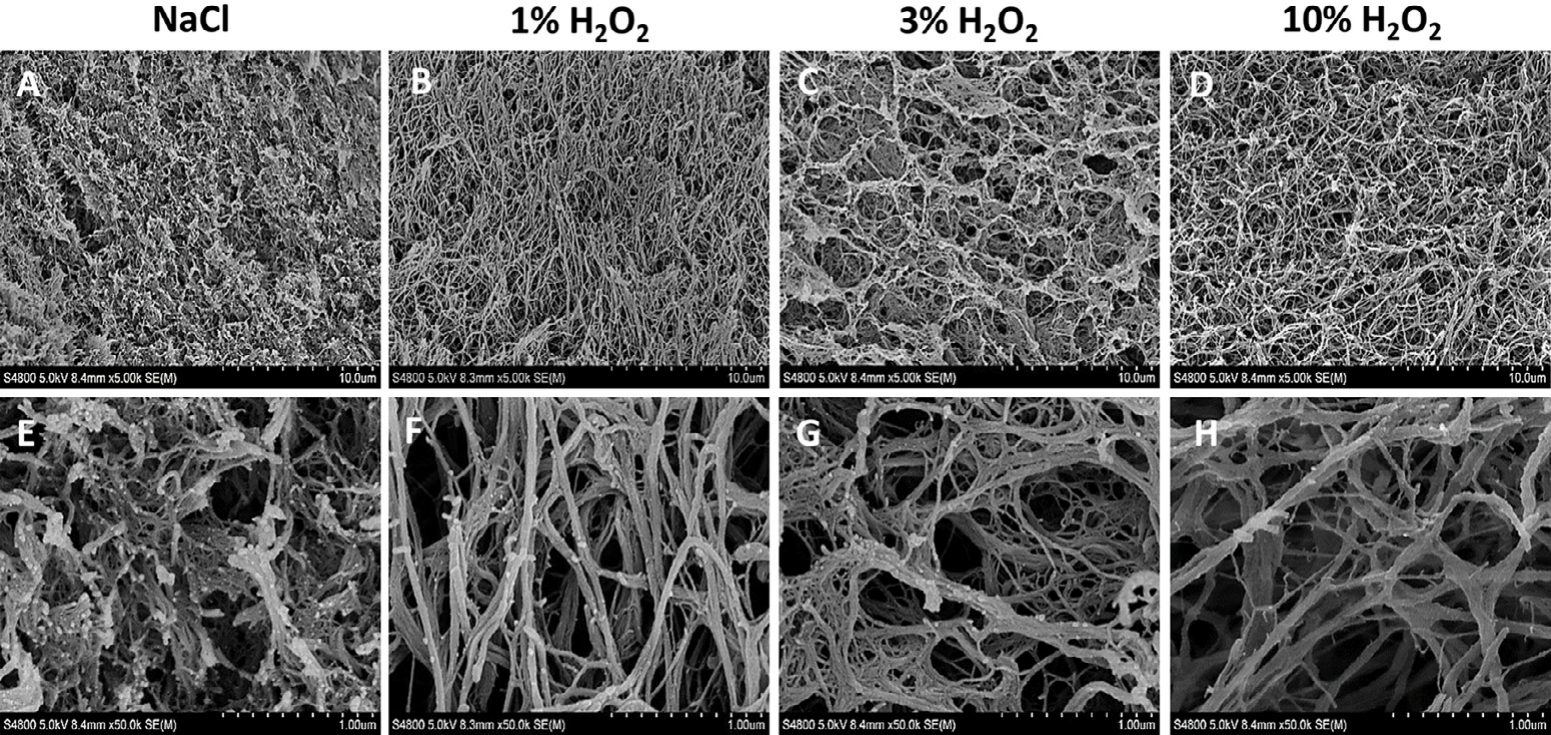
SEM images of fibrin clots immersed in NaCl (Control), 1%, 3% and 10% H_2_O_2_ at 5X (A-D) and 50X magnification (E-H).

The microphotographs show that H_2_O_2_ affects the structure of the fibrin clot in a concentration-dependent manner, correlating with the macroscopic and spectrophotometric results (Figure 5). Compared to the control (NaCl), which is a mesh of tightly packed and cross-linked fibrin fibres (Figure 5A, E), as H_2_O_2_ is added, the mesh opens up and the fibres re-arrange in a less tightly packed manner. The highest concentration of 10% H_2_O_2_ shows a fibrin mesh with a different structure to that of the control (Figure 5D, H). With the increase in H_2_O_2_ concentrations, fibre aggregation is observed. In the control group, the fibres appear thinner than at 1% (Figure 5B, F), 3% (Figure 5C, G) and 10% H_2_O_2_ (Figure 5D, H). The process seems to be homogeneous affecting the entire clot simultaneously.

### Characteristic features of the fibrin network

**Figure 6:**
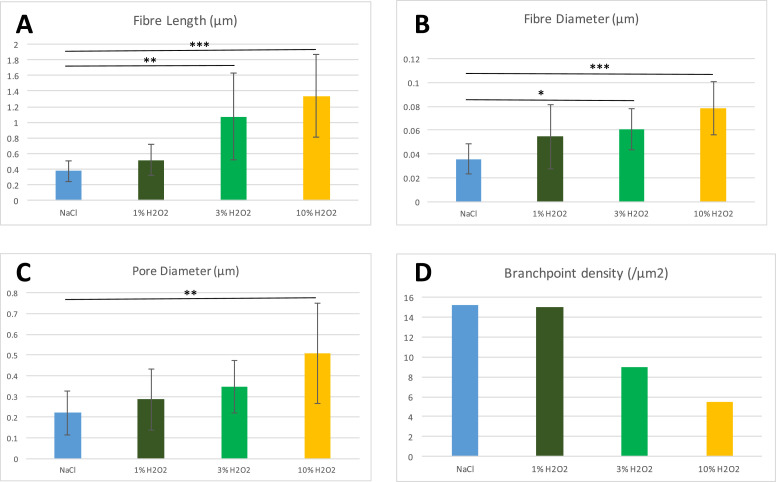
Fibrin fibre length (A) and diameter (B), with pore diameter (C) and branch point density (D) following immersion in NaCl (Control), 1%, 3% and 10% H_2_O_2_. Results expressed as a mean ± SD. p-values * <0.05, **<0.01, ***<0.001.

H_2_O_2_ affects the clot microstructure by increasing the length and diameter of the fibres (Fig, 6A, B), whilst decreasing fibre branching (Figure 6D) in a dose-dependent manner. However, the observed reduction in branchpoint density was not statistically significant. Fibre length increased from a mean of 375 nm (±128 SD) in the control group to 1338 nm (±538 SD) in the 10% H_2_O_2_ group together with an increase in fibre diameters from 36 nm (±13 SD) to 78 nm (±22 SD), although there was a wide variation in individual fibre sizes in all samples. This meant that the average fibre in the 10% H_2_O_2_ sample was 30 monomers long and 9-13 monomers thick, based on the 45 nm length and 6–9 nm width of a fibrinogen molecule measured by Hall and Slayter (1959),^24^ and Estis and Haschemeyer (1980).^25^ The reduction in branchpoint density (Figure 6D) was also accompanied by larger pore sizes (Figure 6C) as the structure opened with the increase in the strength of H_2_O_2_.

## Discussion

Our study provides a possible biochemical mechanism for the anecdotal clinical observation that H_2_O_2_ is an effective agent for removing clots and dried-on blood during surgical procedures. To provide enough sample material for both microstructure and macroscopic volumetric analyses, the fibrin degradation model for this study used fibrin clots with a starting volume of 5mm^2^, whereas physiological fibrin clots are a fraction of a millimetre in size. The differences in surface area to volume ratio between our in vitro model and physiological fibrin clot may account for the differences in fibrinolysis timings found for the in vitro (75-90 minutes) versus the observed in vivo (seconds – minutes) effect. What we are able to deduce from our in vitro model is that the mechanism appears to be time and dose dependent and this supports a potential change in practice for leaving any 3% H_2_O_2_ *in situ* for a period of time. In practice, this could be done via a gauze swab soaked in H_2_O_2_ and left over the area before attempting to remove any dried-on blood rather than straight away. The time for maximal H2O2 effect in vivo warrants further investigation.

Our SEM data suggest a mechanism of action for the breakdown of a fibrin clot by H_2_O_2_ involving the quaternary structure of the clot. Normally, a fibrin clot consists of a mat-like arrangement of fibrils which traps erythrocytes in an otherwise insoluble structure. Instead, we observed major changes to the structure as the concentration of H_2_O_2_ was increased. Initially, the fibrin clot has a typical mat-like structure (saline only), changing to a more individual (but loosely knit) arrangement following exposure to 1% H_2_O_2_. At the highest concentrations of H_2_O_2_, we observed an even looser and more porous structure. As no enzymes were introduced during our experiment, we have concluded that the active agent responsible for these changes was the H_2_O_2_ itself. We speculate that H_2_O_2_ has a direct effect on cross-linking and potentially has effects on the tertiary structure of the fibrin monomers making up the clot. Long fibre lengths are generally accompanied by large fibre diameters (Figure 5), in keeping with what has previously been described by Baradet et al (1995).^26^ Branching becomes less frequent under conditions that promote lengthwise protofibril growth, which may be due to the accumulation of weak noncovalent interactions along the long protofibrils.^27^ We noticed similar changes in our own experiments (Figures 5 and 6). Our data suggest that branching and lateral aggregation compete^27^ and the latter therefore results in thicker and longer fibres, with fewer branch points and greater pore sizes with increasing concentrations of H_2_O_2_. As a powerful oxidising agent, it is logical that the ability of H_2_O_2_ to form hydrogen bonds with adjacent monomers will affect clot structure. Although it was beyond the scope of this study, it might be of interest to examine the effects of H_2_O_2_ on fibrin protein structure in greater detail using atomic force microscopy, and to perform rheological analysis to elucidate the effects of these structural changes on fibrin clot viscoelastic properties.

H_2_O_2_ is a key mediator of normal wound healing processes, contributing to paracrine signalling processes that recruit leukocytes to evoke an initial proinflammatory response,^28^ and drive the production of new tissue through VEGF, COX-2 and EGFR signalling^29^, ^30^, ^31^ and wound remodelling via TGFβ1.^32^ Despite in vitro studies indicating potential metabolic and genotoxic effects of applied hydrogen peroxide on host cells owing to the generation of reactive oxygen species^8^, ^9^, ^10^ as well as a suggestion that H_2_O_2_ promotes embryonic fibroblast proliferation contributing to the production of scar tissue^33^ no in vivo deleterious effect on wound healing^30^^,^^34^ or skin graft donor site healing^35^ has been found. In fact, 5 minute application gauze soaked in 2% H_2_O_2_ actually increased skin graft take in chronic-colonised burn wounds in a clinical trial.^36^ The promotion of a beneficial healing environment needs to be balanced with the potentially genotoxic sequelae of oxidative stress generated by H_2_O_2_ irrigation and induction of clinically relevant DNA damage in exposed host cells warrants further investigation.

A mechanically stable clot is necessary to enable haemostasis, the first step in wound healing, where the aggregation of platelets to fibrinogen occurs via integrin αIIbβ3 receptors. Previous studies have demonstrated the inhibitory effect of H_2_O_2_ on fibrin clot formation.^16^, ^17^, ^18^, ^19^ However, to our knowledge, this is the first study to demonstrate an effect of H_2_O_2_ on established fibrin clots. The effectiveness of H_2_O_2_ in accelerating fibrin breakdown may therefore be of benefit for the removal of other fibrin dependent structures such as wound slough. We continue to use a 3% H_2_O_2_ solution during surgical procedures to irrigate wounds and to assist in the removal of dried-on blood clots. However, we now do so with some insight into the mechanisms that underpin these clinically useful properties of H_2_O_2_.
